# Reversible Atmospheric Water Harvesting Using Metal-Organic Frameworks

**DOI:** 10.1038/s41598-020-58405-9

**Published:** 2020-01-30

**Authors:** Matthew W. Logan, Spencer Langevin, Zhiyong Xia

**Affiliations:** Research and Exploratory Development Department, Johns Hopkins University Applied Physics Laboratory, Laurel, MD 20723 United States

**Keywords:** Materials science, Metal-organic frameworks, Structural properties

## Abstract

The passive capture of clean water from humid air without reliance on bulky equipment and high energy has been a substantial challenge and has attracted significant interest as a potential environmentally friendly alternative to traditional water harvesting methods. Metal-organic frameworks (MOFs) offer a high potential for this application due to their structural versatility which permits scalable, facile modulations of structural and functional elements. Although MOFs are promising materials for water harvesting, little research has been done to address the microstructure-adsorbing characteristics relationship with respect to the dynamic adsorption-desorption process. In this article, we present a parametric study of nine hydrolytically stable MOFs with diverse structures for unraveling fundamental material properties that govern the kinetics of water sequestration in this class of materials as well as investigating overall uptake capacity gravimetrically. The effects of temperature, relative humidity, and powder bed thickness on the adsorption-desorption process are explored for achieving optimal operational parameters. We found that **Zr-MOF-808** can produce up to 8.66 L_H2O_ kg^−1^_MOF_ day^−1^, an extraordinary finding that outperforms any previously reported values for MOF-based systems. The presented findings help to deepen our understanding and guide the discovery of next-generation water harvesting materials.

## Introduction

Despite the rapid growth of modern infrastructure, access to clean water remains a critical issue and challenge to humanity that is projected to increase at a rate faster than that of energy production^1^. Limited access to freshwater due to the absence of sources, such as lakes, rivers, and groundwater is becoming even more problematic, with many of these sources becoming contaminated from human activities. Traditional means to acquire clean water, such as reverse osmosis and distillation, is costly and energy-intensive which in turn restricts real-world uses^2,3^. Therefore, it is essential to find portable solutions that are simple and low cost to produce clean water on demand in various environments.

A substantial effort on accessing nontraditional water reserves, such as atmospheric water vapor, focuses on the ability to supply freshwater on-demand virtually anywhere on the earth^4^. Atmospheric water harvesting offers an attractive alternative by providing access to the omnipresent water vapor in the earth’s atmosphere off the grid and in virtually any environment. Direct water harvesting from air has been demonstrated through cooling water vapor below its saturation pressure is not practical in dry climates due to its high energy demands^3^.

Metal-organic frameworks (MOFs) due to their unique micro-structure, intrinsic porosity, and unprecedented functional and chemical control have a high potential to be used for harvesting water from air^5,6^. Recently, the use of MOFs to leverage the earth’s natural thermal swing process to efficiently sequester clean water with little to no additional energy input has been demonstrated^7^. MOFs are commonly constructed through the self-assembly of metal oxide clusters (also known as nodes) and multifunctional organic linkers (also known as struts), and form highly crystalline complex 3D architectures. The modularity in MOFs allows the properties of these distinct hierarchical structures to be tunable from the molecular level of the inorganic node and/or organic linker to that of a microscopic level and subsequently particle morphology. Recently, Hanikel *et al*. demonstrated that a water generation capability of 1.3 L kg_MOF_^−1^ day^−1^ was achieved by using **Al-MOF-303** at 32% relative humidity (RH) and 27 °C using multiple adsorption-desorption sequences^4^. Even though the water recovery rate is low, their system is a breakthrough in the rapid production of water from the atmosphere using multiple harvesting cycles per day without the need of energy inputs. This work reveals the significance of water uptake dynamics, as opposed to the majority of previous studies that focused on controlling the adsorption equilibrium and total water capacity in materials^8–11^. Although these are essential characteristics for water sequestering materials to possess, the more valuable property is fast and efficient production of water. Consequently, understanding the structure-property relationship controlling these effects is vital for the creation of optimized water harvesting materials based on MOFs.

In this study, we evaluated the effects of relative humidity, temperature, and the dynamic sorption properties on a wide range of MOFs with different nodes and strut functionalities. The structure-property relationship and its role in the water capture and release kinetics are carefully addressed under several well controlled conditions. Our ultimate goal is to develop a design strategy for ambient water harvesting systems and guide the development of next-generation water harvesting materials.

## Results and Discussion

**Figure 1:**
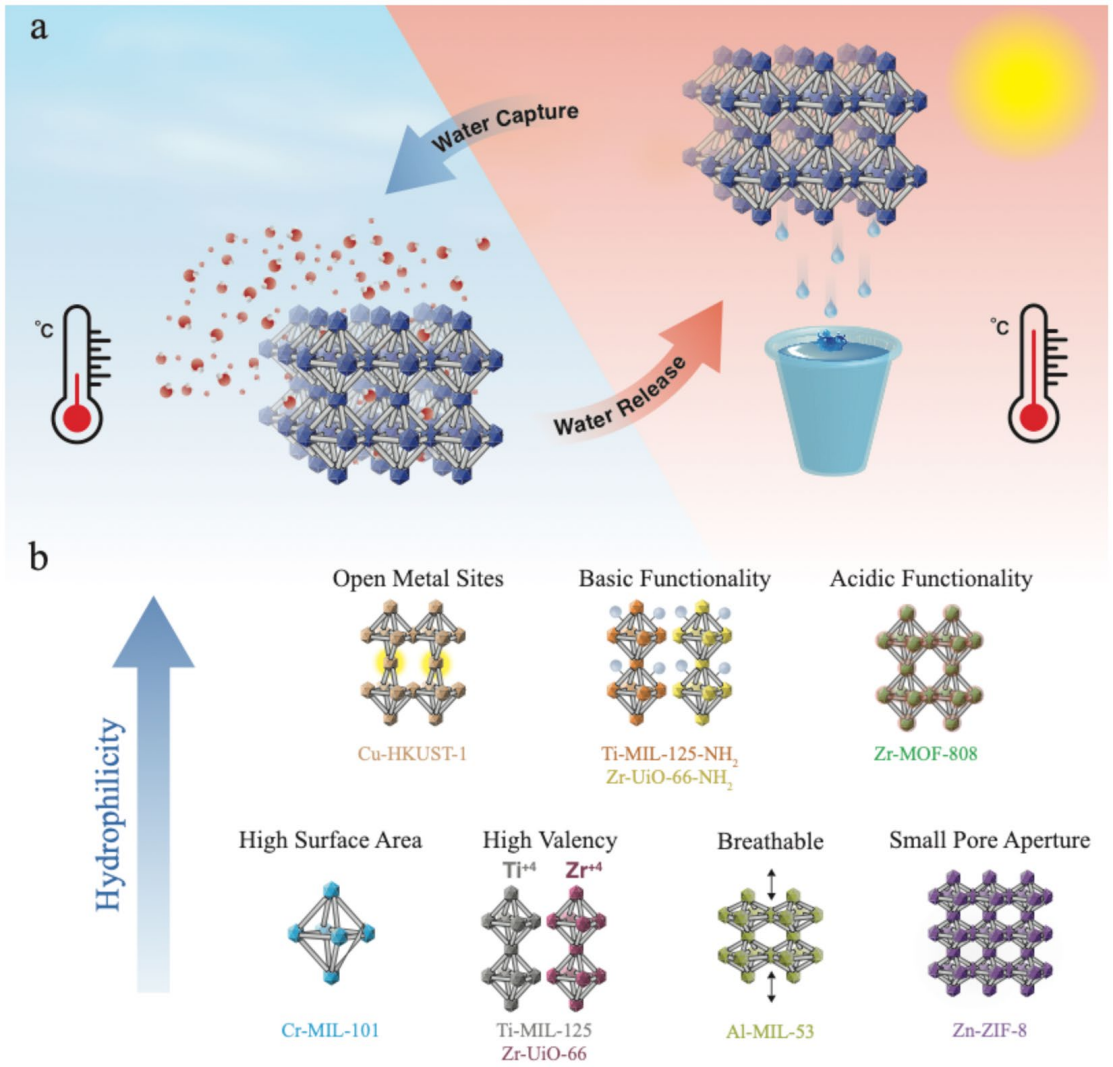
Schematic illustration of the MOF water harvesting system (**a**) and a general overview of MOFs employed in the present study (**b**), highlighting different functionalities and representative MOFs.

The MOF-based water harvesting system is schematically shown in Fig. 1a, which has been simplified to highlight the water harvesting using MOF. Further, the capture and release cycles occur independently in regular cycles using a thermal swing process. Figure 1b shows the simplified morphologies of the MOFs used in this study. The details of the MOF synthesis can be found in the methods section. In-depth characterization of all these MOFs can be found in the Supplementary Information. Briefly, **Al-MIL-53**^12^ has a distinctive breathing capability with moderate accessible surface area; **Ti-MIL-125**^13^, **Zr-UiO-66**^14^, and **Cr-MIL-101**^15^ contain high valence metal-oxide clusters and hard carboxylate-based organic linkers owing to their excellent chemical stability and limited chemical functionality that restricts polarity in the view of this study. **Ti-MIL-125-NH**_**2**_^16^ and **Zr-UiO-66-NH**_**2**_^14^ are the respective isoreticular motifs to **Ti-MIL-125** and **Zr-UiO-66** with polar and basic functionalities substituted on the diatopic organic linker. **Zr-MOF-808** possesses an acidic pore and high surface area^17^. **Zn-ZIF-8** contains polar properties but with narrow pore apertures (3.4 Å) that exhibit hydrophobic properties^18^. **Cu-HKUST-1** is constructed by a tritopic organic moiety and copper paddle-wheel secondary building units (SBUs) with open metal sites^19^.

**Figure 2:**
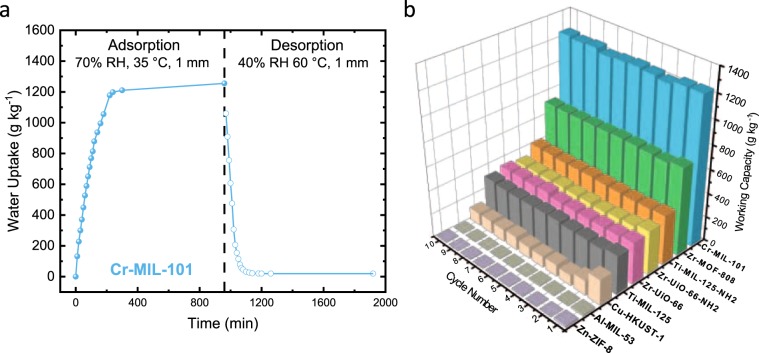
Water sorption properties of MOFs studied in present work. (**a**) Dynamic water vapor adsorption and desorption of **Cr-MIL-101** in loose powder bed with a packing density of 0.65 at 1 mm thickness. Adsorption was performed at 70% RH in solid spheres and 35 °C, desorption at 30% RH and 60 °C and shown in open spheres. (**b**) Working capacities of studied MOFs over ten consecutive sorption cycles. Adsorption cycles were performed at 70% RH over 24 h at 22 °C; desorption cycles were performed at 60 °C for 24 h under ambient humidity of (~30%). **Zn-ZIF-8** (lavender), **Al-MIL-53** (pickle), **Cu-HKUST-1** (brown), **Ti-MIL-125** (black), **Zr-UiO-66** (pink), **Ti-MIL-125-NH**_**2**_ (orange), **Cr-MIL-101** (blue), **Zr-MOF-808** (green), and **Zr-UiO-66-NH**_**2**_ (yellow).

To simulate the real world conditions, the maximum amount of harvested water is not determined by the uptake equilibrium, but rather by the amount of the water that can be desorbed after saturation. In this work, the maximum harvested water is referred to as working capacity (g_H2O_ kg^−1^_MOF_) and should be the same as the amount of water captured. This working capacity is related to the enthalpy of desorption, e.g., how much energy is needed for removing the physisorbed molecules from the MOF. Ideally, this desorption enthalpy should be high enough to allow water adsorption at low RH and remain bound until prescribed conditions. The desorption process should require little energy input and release all of the adsorbed water. To study this behavior, ten adsorption and desorption cycles were performed using the as-prepared MOFs, and the results are shown in Fig. 2. The adsorption cycle was performed on activated MOFs over 24 h at 70% RH and 22 °C. In comparison, the desorption was performed at 30% RH and 60 °C to simulate conditions that can easily be achieved for a thermal-mediated desorption process with little to no energy input.

The gravimetric uptake capacity at equilibrium for water vapor has a strong correlation with total pore volume and is remarkably similar to previously reported data obtained using water vapor isotherm, with exception to **Zn-ZIF-8** (Fig. 2, Table 1, and Supplementary Figs. S10–S18)^20^. Water recovery in **Zn-ZIF-8** and **Al-MIL-53** show recoveries of less than 6 and 2 g_h2o_ kg^−1^_MOF_ at the 10^th^ cycle, respectively. This performance comes from the hydrophilic nature and inability to uptake water vapor in both MOFs. **Cu-HUKST-1** shows decreased working capacities over the first 3 cycles from 212 to 94 g_H2O_ kg^−1^_MOF_. This is significantly less than the 487 g_H2O_ kg^−1^_MOF_ that the MOF adsorbed within the timeframe for the measurement. This finding shows that during the first desorption cycle, less than 50% of the initial adsorbed water can be removed under mild desorption conditions. This partial saturation after desorption is consistent with a strong interaction between water and the open Cu metal sites that cannot easily be removed under the desorption protocol of 60 °C and 30% RH. Similar strong interactions have also been observed in silica gels^21^ and zeolites^22^ which require elevated temperatures, in some cases in excess of 300 °C, to remove water effectively. This amount of energy to regenerate a saturated material is not effective energetically or kinetically and can even cause decomposition.

**Table 1:** Summary MOFs studied with N_2_ gas for determining water vapor sorption properties.

MOF	SA^*a*^ (m^2^ g^−1^)	V_p_^*b*^ (cm^3^ g^−1^)	Uptake capacity ^*c*^ (g kg^−1^)	Working capacity^*d*^ (g kg^−1^)	Water desorbed (%)
Ti-MIL-125	1153	0.47	323	313	97
Ti-MIL-125-NH_2_	1358	0.55	413	409	99
Zr-UiO-66	959	0.40	347	338	97
Zr-UiO-66-NH_2_	1109	0.46	364	355	98
Zr-MOF-808	1880	0.69	744	714	96
Cr-MIL-101	2579	1.63	1263	1246	98
Cu-HKUST-1	1512	0.41	218	105	48
Al-MIL-53	814	0.41	13	2	15
Zn-ZIF-8	1835	0.69	15	6	40

^a^Surface area calculated from Brunauer-Emmett-Teller (BET) model analysis of N_2_ gas adsorption-desorption isotherm at 77 K. ^b^Pore volume calculated at *p*/*p*_*o*_ = 0.4 of N_2_ adsorption isotherm (77 K). ^c^Water vapor uptake capacity was determined gravimetrically; the value is taken from the first cycle adsorption at 70% RH and 22 °C at ambient pressure. ^d^Working capacity was determined gravimetrically by the difference in the amount of water desorbed and adsorbed during water cycle stability and recovery studies.

The high-valence **Ti-MIL-125** (246 g_H2O_ kg^−1^_MOF_) and **Zr-UiO-66** (338 g_H2O_ kg^−1^_MOF_**)** show similar working capacities to their isoreticular counterparts, i.e., **Ti-MIL-125-NH**_**2**_ (393 g_H2O_ kg^−1^_MOF_) and **Zr-UiO-66-NH**_**2**_ **(**320 g_H2O_ kg^−1^_MOF_). The highest working capacity was observed in **Zr-MOF-808** and **Cr-MIL-101** of 616 and 1187 g_H2O_ kg^−1^_MOF_, respectively. Comprehensively, these working capacity results agree with the analogous pore volumes and BET surface areas in each respective MOF. Furthermore, the water vapor recovery remained unaltered throughout ten cycles. This outstanding resilience was confirmed with post-cycled powder x-ray diffraction (PXRD) analysis, which displays that there is no structural change in post-cycled MOFs (Supplementary Figs. S1–S9). There is no apparent trend in uptake capacities and surface functionalities.

Owing to their superior working capacities, **Ti-MIL-125**, **Ti-MIL-125-NH**_**2**_**, Cr-MIL-101**, **Zr-MOF-808**, and **Zr-UiO-66-NH**_**2**_ were further investigated using a carefully controlled parametric time study for understanding the water vapor adsorption-desorption processes. This can lead to further insight into the dynamic properties responsible for controlling water mass-transport and diffusivity coefficients. The diffusional behavior of water in particular materials is RH dependent and fluctuates with temperature making a comprehensive study difficult. Furthermore, the presence of heat transfer and more complex diffusional phenomenon often lead to spurious coefficients that represent a bulk average of the materials’ intrinsic properties. For this purpose, we have limited our study to a thin-layer, loose grain powder bed reactor under high RHs to ensure reproducibility and relevance to practical applications. The reactor geometry, sorbent mass used, bed thickness, packing porosity, and crystallite sizes were all carefully controlled.

**Figure 3:**
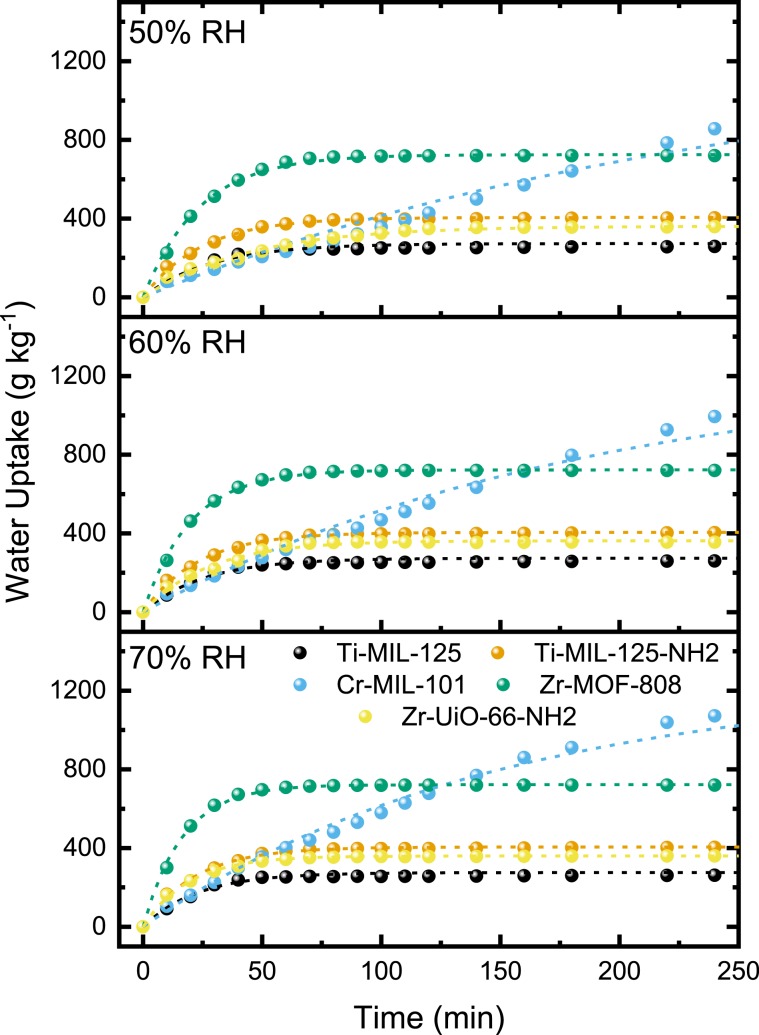
Water adsorption on loose powder bed samples with a packing porosity of ~ 0.65–0.7 and sample depths of 1 mm at 22 °C and 50, 60, and 70% RH. MOF samples were fully activated prior to the adsorption kinetics study. Experimental results are shown as solid spheres, and LDF fitted plot as a dashed line in representative color. **Ti-MIL-125** (black), **Ti-MIL-125-NH**_**2**_ (orange), **Cr-MIL-101** (blue), **Zr-MOF-808** (green), and **Zr-UiO-66-NH**_**2**_ (yellow).

MOFs were compared by exposing them to various conditions over a 4000 min duration. The adsorption of water is recorded gravimetrically and plotted against time (Figs. 2a and 3). The water uptakes exhibited an apparent 1^st^ order behavior which can be modeled using linear driving force (LDF) and fitted accordingly with R^2^ > 0.95 in all accounts when modeled over the entire data range (Supplementary Figs. S38–S48, Table 2, and Supplementary Tables S1–S5)^23^. LDF offers analytical simplicity by stating the average sorbate uptake rate is determined by the products of the amount required to reach equilibrium and the rate constant.

1 $$\frac{d{M}_{t}}{dt}=k({M}_{e}-{M}_{t})$$

where *M*_*t*_ (g_h2o_ kg^−1^_MOF_) is the amount of water vapor at time *t*, M_*e*_ (g_h2o_ kg^−1^_MOF_) is the equilibrium uptake, and *k* (min^−1^) is the rate constant. This implies when plotting ln(M_*e*_-M_*t*_) M_*e*_^−1^ versus time (*t*) the LDF mass-transfer coefficient can be obtained. It was described previously that the initial rate of the adsorption and desorption process, R_0_, is accurate at describing gravimetric water uptake at any given time, providing a numerical value to assess dynamic behavior^4^. Morphological and crystallite size information was determined using scanning electron microscopy (SEM) (Supplementary Figs. S27–S32) and dynamic light scattering (DLS) (Supplementary Figs. S33–S37), which was used to calculate water vapor intracrystalline diffusivity parameters based on the mass-transfer coefficients with the assumption that intercrystalline diffusivity being the rate-limiting step. *D*_*H2O*_ can be calculated using the formula^24^:

2 $$\,{{D}}_{{H2O}}=\frac{k{{R}_{MOF}}^{2}}{15}$$

where *D*_*H2O*_ (cm^2^ min^−1^) is the intercrystalline diffusivity coefficient, *R*_*MOF*_ (cm) is the average radius of MOF crystallites, and *k* (min^−1^) from Eq. 2.

**Table 2:** LDA fitting of experimental water vapor adsorptions results of variable RH on tested MOFs^a^.

MOF	50% RH			60% RH			70% RH		
	R_0_ (g kg^−1^ min^−1^)	*k* (min^−1^)	*D*_*H2O*_ (cm^2^ min^−1^)	R_0_ (g kg^−1^ min^−1^)	*k* (min^−1^)	*D*_*H2O*_ (cm ^2^ min^−1^)	R_0_ (g kg^−1^ min^−1^)	*k* (min^−1^)	*D*_*H2O*_ (cm^2^ min^−1^)
Ti-MIL-125	7.15	2.50 × 10^−2^	1.39 × 10^−4^	7.86	2.74 × 10^−2^	1.52 × 10^−4^	8.65	3.02 × 10^−2^	1.68 × 10^−4^
Ti-MIL-125-NH_2_	1.69 × 10	3.77 × 10^−2^	5.82 × 10^−4^	1.82 × 10	4.07 × 10^−2^	6.29 × 10^−4^	1.98 × 10	4.42 × 10^−2^	6.84 × 10^−4^
Zr-UiO-66-NH_2_	6.56	2.27 × 10^−2^	2.58 × 10^−5^	1.50 × 10	3.95 × 10^−2^	5.90 × 10^−5^	1.72 × 10	4.52 × 10^−2^	6.67 × 10^−5^
Zr-MOF-808	3.17 × 10	4.25 × 10^−2^	1.80 × 10^−3^	3.72 × 10	5.64 × 10^−2^	2.12 × 10^−3^	4.74 × 10	7.20 × 10^−2^	2.70 × 10^−3^
Cr-MIL-101	3.71	4.02 × 10^−3^	4.42 × 10^−6^	5.58	4.44 × 10^−3^	6.65 × 10^−6^	7.97	6.35 × 10^−3^	9.50 × 10^−6^

^a^Loose powder bed samples with a packing porosity of ~0.65–0.7 and sample depths of 1 mm at 22 °C. The average particle size was used to calculate the diffusional coefficient.

The dynamic water uptake was studied in triplicate for free bed samples with packing porosity of ~0.65–0.7 and bed depths of 1 mm at 22 °C and 70% RH (Fig. 3 and Supplementary Fig. S38). Table 2 shows the results of LDF fitting and shows that the MOFs with polar functionalities uptake water significantly faster, exhibiting initial rates of 47.4, 19.8, and 17.2 g kg^−1^ min^−1^ for **MOF-808, MIL-125-NH**_**2**_, and **UiO-66-NH**_**2**_, respectively. More nonpolar **Ti-MIL-125** and **Zr-MIL-101** show decreased uptake rates of 8.65 and 7.97 g kg^−1^ min^−1^, respectively. Impressively, all MOFs reached saturation in 60 min, except for **Cr-MIL-101**, which took more than 4 h. It is interesting to observe that while constraining the powder bed thickness to 1 mm, the adsorption uptake dynamics are dominated by polar functionality and not the accessible surface area as **Cr-MIL-101** has an uptake almost double of that of **Zr-MOF-808** but exhibits an initial rate that is almost six times slower. As expected, when RH is incrementally decreased to 50%, reduction in initial rates, adsorption kinetics, and diffusivities with minor deviations in saturation equilibriums was observed. Furthermore, the relative trends within each humidity trial between each MOF remain comparable.

**Figure 4:**
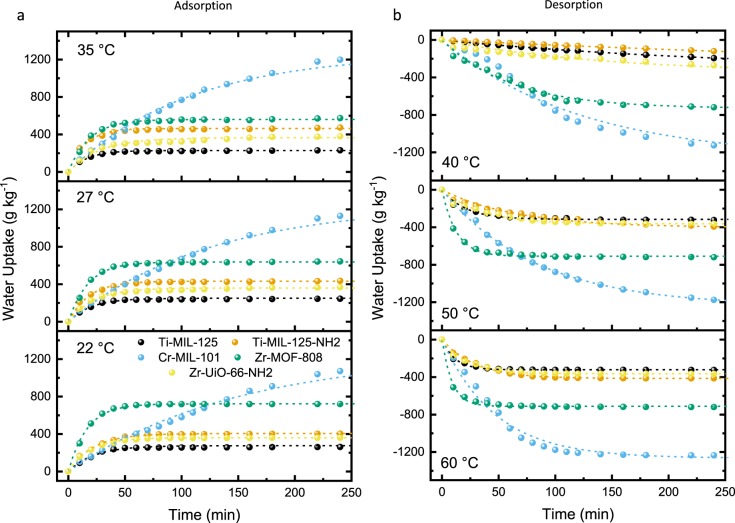
Thermal dependence of water vapor dynamics on fixed powder bed samples with packing porosities of ~0.65–0.7 and sample depth of 1 mm. (**a**) Water vapor adsorption at 70% RH and 22, 27, and 35 °C and (**b**) water vapor desorption at 30% RH and 40, 50, and 60 °C. Experimental results are shown as solid spheres, and LDF fitted plot as a dashed line in representative color. **Ti-MIL-125** (black), **Ti-MIL-125-NH**_**2**_ (orange), **Cr-MIL-101** (blue), **Zr-MOF-808** (green), and **Zr-UiO-66-NH**_**2**_ (yellow).

We also found that ambient temperature played an important role on both adsorption and desorption, which in turn affected water vapor interactions and the physisorption process (Fig. 4, Table 3, and Supplementary Table S2). The investigation into the intracrystalline diffusivity barriers of water vapor adsorption process on MOFs activation energies was calculated at 70% RH using the Arrhenius equation generated from data collected at 22, 27, and 35 °C. The results show that for more hydrophobic MOFs (**Ti-MIL-125** and **Cr-MIL-101**), the uptake mass-transfer coefficient and initial rate are enhanced with increased temperature as expected. In contrast, more polar MOFs (**Ti-MIL-125-NH**_**2**_, **Zr-UiO-66-NH**_**2**_, and **Zr-MOF-808**) experienced a decrease in their rate constants at elevated temperatures. The latter can be attributed to two key reasons: (1) the enhanced mass-transfer rates from the diffusion of water vapor prompted by elevated temperature; (2) the attractive interactions responsible for the physisorption of water adsorption is exothermic and effectively weakened as temperature increases and facilitates desorption^25^. Consequently, adsorption kinetics are negatively affected by the increase in temperature. This becomes more evident in MOFs that have strong interactions with water vapor. In short, MOFs with polar functional groups and high enthalpy of adsorption, e.g., more hydrophilic MOFs, tend to have a more negative dependence on temperature than those with a lower enthalpy of adsorption regardless of pore size, volume, and accessible surface area (**Ti-MIL-125** and **Cr-MIL-101)**. Interestingly, **Ti-MIL-125-NH**_**2**_ and **Zr-UiO-66-NH**_**2**_ have similar available surface areas, water uptakes, metal-ions, and linker properties: but have a significant difference in the activation energies of −8.17 and −31.7 kJ mol^−1^, respectively. This variance in hydrophilicity can also be observed in the influx point of 24 and 16% RH (Supplementary Table S1) respectively for **Ti-MIL-125-NH**_**2**_ and **Zr-UiO-66-NH**_**2**_, exhibiting that the latter is more hydrophilic in nature. This temperature dependence is an important finding that cannot be determined from water vapor adsorption isotherms alone and emphasizes the importance of time-dependent uptake studies to help in materials design. There was no appreciable impact from temperatures on the equilibrium reached, excluding for **Zr-MOF-808**, decreasing from 742 to 560 g kg^−1^ for 22 and 35 °C, respectively.

**Table 3:** The rate dependence and activation energies of water uptake at 70% RH^a^.

MOF	R_0_ (g kg^−1^ min^−1^)		E_a_^*b*^ (kJ mol^−1^)	R^2^_adj_
	22 °C	35 °C		
Ti-MIL-125	8.65	1.02 × 10	9.62	0.999
Ti-MIL-125-NH_2_	1.98 × 10	1.72 × 10	−8.17	0.988
Zr-UiO-66-NH_2_	1.72 × 10	9.82	−31.7	0.949
Zr-MOF-808	4.74 × 10	2.60 × 10	−34.1	0.966
Cr-MIL-101	7.97	9.87	12.3	0.978

^a^Loose powder bed samples with a packing porosity of ~0.65–0.7 and sample depths of 1 mm. ^b^Activation energy calculated from the slope from the Arrhenius plot of the kinetic rate constants vs. temperatures.

The subsequent desorption was performed after samples were equilibrated at 70% RH and 22 °C over 24 hours (Fig. 4b and Supplementary Figs. S46–S48). Studies were completed at 30% RH and temperatures of 40, 50, and 60 °C. There is no apparent trend in the desorption rates, with respect to porosity or functionality, of all MOF that show initial desorption rates ranging from 0.632–10.8 g kg^−1^ min^−1^ at 40 °C to 16.7–74.5 g kg^−1^ min^−1^ at 60 °C. **Zr-MOF-808** exhibits the most attractive dynamic desorption properties under every condition studied.

The maximum potential water production can be estimated from the combination of the dynamic adsorption and desorption data (Table 4). It should be noted that the presented data assumes that collection efficiency is 100% and that the relative humidity changes with the adsorption and desorption cycle. The numbers represent an ideal number that is not representative of what is possible, but it provides an upper limit to what is potentially achievable with intelligent heating and chilling to modulate dew point, water desorption from saturated sorbent, and water condensation. Furthermore, this module, when used in conjunction with a fan to facilitate water vapor diffusion, will be energy-intensive and requires appropriate balancing with available energy sources that could be farmed with photovoltaics or other means. Surprisingly, the nonpolar **Ti-MIL-125** has the potential to harvest up to 3.96 L_H2O_ kg^−1^_MOF_ day^−1^, almost half that of the acidic-functionalized **Zr-MOF-808** (8.66) and similar to the amino-functionalized **Ti-MIL-125-NH**_**2**_ (4.17) and **Zr-UiO-66-NH**_**2**_ (3.97). **Cr-MIL-101** can generate up to 1.67 L_H2O_ kg^−1^_MOF_ day^−1^, only being able to be cycled 1.3 times per day. This is significantly smaller than that of **Ti-MIL-125**, which is noteworthy, given it has over four times the working capacity and shows a maximum daily water output of less than half. It is important to point out that the proper selection of materials is dependent upon environmental constraints such as the RH and ability to harvest energy. If there is little available energy, there is a restriction on the number of cycles that can be facilitated by additional peripherals to aid in heating, cooling, and circulating air.

**Table 4:** Water recovery potential of MOFs evaluated^a^.

MOF	Working Capacity (g kg^−1^)	Cycle time^*b*^ (min)	Recovery potential (L kg^−1^ day^−1^)
Ti-MIL-125	275	100	3.96
Ti-MIL-125-NH_2_	405	140	4.17
Zr-UiO-66-NH_2_	359	130	3.97
Zr-MOF-808	722	120	8.66
Cr-MIL-101	1252	1080	1.67

^a^Water adsorption at 22 °C, 70% RH, packing porosities of ~0.65–0.7, and sample depth of 1 mm. Water desorption at 60 °C, 30% RH, packing porosities of ~0.65–0.7, and sample depth of 1 mm. ^b^Cycle time calculated from the sum of the time it takes for complete saturation during adsorption and the time for complete desorption for respective MOF.

**Figure 5:**
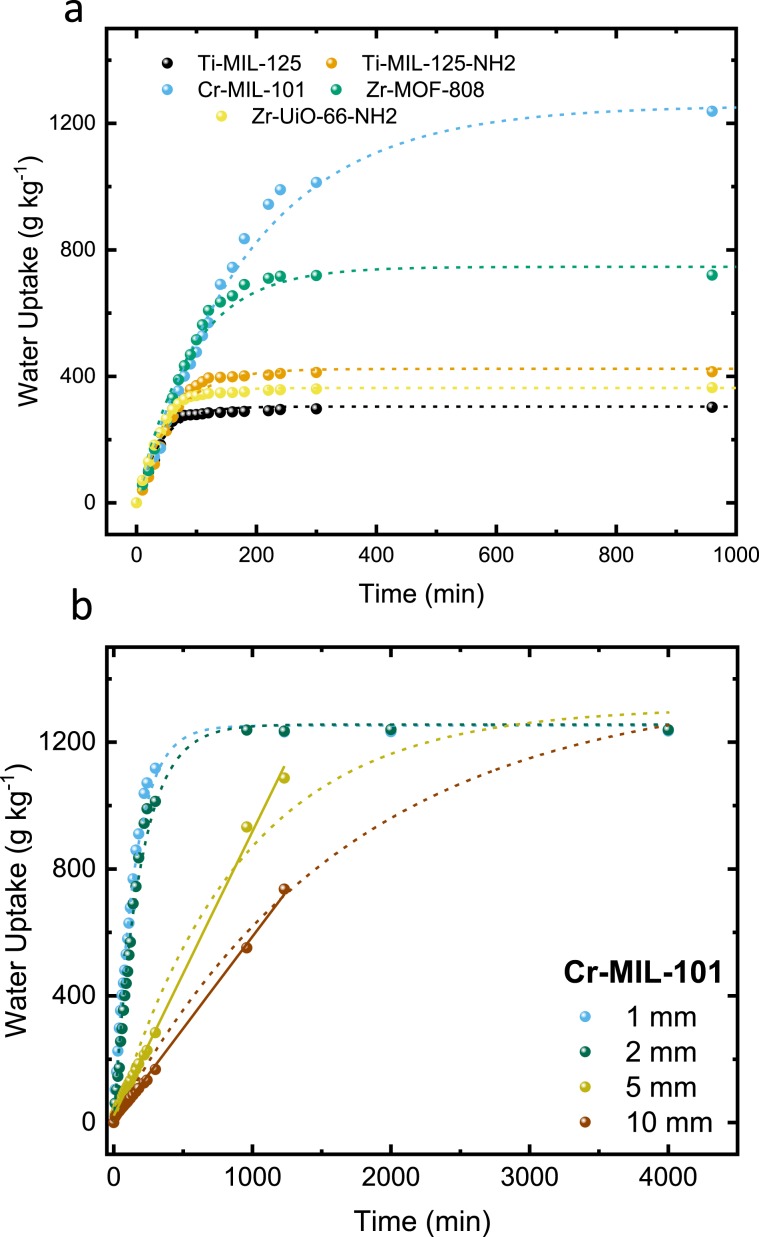
Exposed surface-area-to-volume ratio dependence of water vapor dynamics on fixed powder bed samples at 70% RH and 22 °C. (**a**) Water vapor adsorption of studied MOFs with a packing porosity of ~0.65–7 and a sample depth of 2 mm. **Ti-MIL-125** (black), **Ti-MIL-125-NH**_**2**_ (orange), **Cr-MIL-101** (blue), **Zr-MOF-808** (green), and **Zr-UiO-66-NH**_**2**_ (yellow). (**b**) Water vapor adsorption of **Cr-MIL-101** at a packing porosity of ~0.65–7 and sample depth of 1 (blue), 2(green), 5 (yellow), and 10 (red) mm. Experimental results are shown as solid spheres, LDF fitted plot as a dashed line, and pseudo-zero order fitted as a solid line in representative color.

Loose-grain powder bed reactors are simple but can suffer from limited diffusivity as bed thickness increases. The role of intracrystalline diffusivity is an import factor in understanding water uptake. For practical considerations, there needs to be a balance between total additional energy, sorption kinetics, and volumetric constraints on the specific system to be employed. The inter- and intra-crystalline mass transport, and by extension intracrystalline diffusivity, strongly influence the adsorption-desorption properties (disregarding thermal parameters) in loose-grain powder beds. Furthermore, if there is limited environmental energy that can be harvested to power thermal swings and circulation fans, the number of cycles needs to be optimized accordingly. The diffusional time scale (*t*) and intercrystalline diffusion (D_intrer_) are greatly affected by sample bed thickness (H_MOF_), as *t* ~ H_MOF_^2^ D_intrer_^−1^^7^. Adsorption dynamics collected at 70% RH and 22 °C for the studied MOFs with packing porosities of ~0.65–0.7 and a sample depth of 2 mm are presented in Fig. 5a and Table 5. Sample depths of 1 mm are primarily controlled from the intrinsic properties of individual crystallites; however, as sample depths are increased to 2 mm, and presumably beyond, all studied MOFs have similar initial rates of uptake. This regime is no longer dominated by specific morphology and functionality; uptake dynamics are determined by intracrystalline diffusivity, with intercrystalline diffusivities being negligible. The most critical material property to consider is the adsorption capacity at equilibrium. Consequently, **Cr-MIL-101** was also studied at bed depths of 5 and 10 mm (Fig. 5b). The 1^st^ order LDA can no longer adequately model the uptake kinetics for **Cr-MIL-101** at these depths and a pseudo-zero models appears to be a better model. This region is no longer kinetically favorable, needing over 24 h to reach equilibrium in both scenarios. Design of a powder bed system that operates under similar conditions as used in this study would require stacked shelves with laminar flow to optimize volumetric uptake capacity such has been recently demonstrated^4^. Regardless, these findings illustrate the significance of powder bed thickness on materials selection, which is primarily driven by the chemical functionality and structural topology. We would like to point out that sample thickness will be affected by thermodynamic factors, e.g., heat generation and dissipation, that can both affect the sorbent material’s performance. As sample thickness increases, the intracrystalline effects will become a more dominant influence that limits the adsorption and desorption kinetics. We see that under our testing conditions there is limited dependence on the specific properties of the MOFs as the depth is increased.

**Table 5:** Rate dependence of water uptake on powder bed depth at 70% RH and 22 °C.

MOF	R_0_ (g kg^−1^ min^−1^)	
	depth = 1 mm	depth = 2 mm
Ti-MIL-125	8.65	7.11
Ti-MIL-125-NH_2_	1.98 × 10	7.58
Zr-UiO-66-NH_2_	1.72 × 10	9.68
Zr-MOF-808	4.74 × 10	7.45
Cr-MIL-101	7.97	6.69

## Conclusion

We investigated a series of MOFs with a wide variety of structural topologies and chemical functionalities for atmospheric water harvesting and studied their structure-property relationships. This work presents the first quantitative direct comparative investigation of MOFs evaluating different simulated operational conditions to facilitate proper materials selection to optimize materials and system design. Interestingly, thermal studies revealed that hydrophilic MOFs have a negative dependence with increasing temperature whilst nonpolar MOFs show enhanced uptake over the same temperature regime. Modulation of powder bed depth suggests the competition between intracrystalline and intercrystalline diffusivities control water vapor dynamics in different thickness regimes, revealing that all MOFs studied have similar uptake dynamics at a bed thickness of 2 mm, regardless of MOF properties. At a powder bed thickness of 1 mm, the role that surface functionality and topology have on water harvesting is significant, showing the performance of **Zr-MOF-808** is superior in most scenarios and, under ideal conditions, could generate up to 8.66 L_H2O_ kg^−1^_MOF_ day^−1^. Unfortunately, **Zr-MOF-808** has an influx point at 36% RH, which limits its applications in arid environments with relative humidities typically ranging from 10–30%. The results presented here enhance our understanding of MOFs as solid-state sorbent materials and of the role that the structure-property relationship plays in dictating materials selection. In our future work, we plan to investigate additional MOFs with similar properties to **Zr-MOF-808**, e.g., those with low RH influx points, high surface areas, and polar functional properties for investigation towards arid conditions. Furthermore, studying MOFs in different geometries such as pellets or extruded in monolithic honeycomb structures, from a volumetric constraint, optimizing thermodynamic properties, as a composite, or even as host matrixes for deliquescent materials, may allow for enhanced uptake performance while maintaining a fixed shape and size. All these are interesting and worth investigating in future work. These will be reported in future reports.

## Methods

### Materials

Chromium (III) nitrate nonahydrate (Cr(NO_3_)_3_•9H_2_O, ≥99%), iron(III) nitrate nonahydrate (Fe(NO_3_)_3_•9H_2_O, ≥98%), titanium (IV) isopropoxide (Ti(O^*i*^Pr)_4_, 97%), zirconium(IV) chloride (ZrCl_4_, ≥99.5%), aluminum nitrate nonahydrate (Al(NO_3_)_3_•9H_2_O, ≥98%), zinc nitrate hexahydrate (Zn(NO_3_)_2_•6H2O, 98%) were purchased from Sigma-Aldrich. The organic linker moieties: 1,4-benzenedicarboxylic acid (H_2_BDC, 98%), 2-amino-1,4-benzenedicarboxylic acid (H_2_BDC-NH_2_, 99%), 1,3,5-benzenetricarboxylic acid (H_3_BTC, 95%), and 2-methylimidazole (HMIM, 99%) were obtained from Sigma-Aldrich. Acid modulation was performed using benzoic acid (HBC, 99.5%) and nitric acid (HNO_3_, 70%). Ultra-high purity He and N_2_ gas (99.999%) were purchased from Airgas. Anhydrous solvents (MeOH and DMF) were purchased from Sigma-Aldrich in Sureseal bottles. Unless specified otherwise, all reactions were performed under ambient laboratory conditions, and all reagents were used as received with no further purification from a commercial source.

### Materials characterization

Powder X-ray diffraction (PXRD) was performed under ambient conditions on a PANalytical Empyrean Series 2 diffractometer in a Bragg-Brentano geometry using a Ni-filtered lined-focused CuK_α_ radiation (λ = 1.54 Å). Data was collected from 5–40 2*θ*-degrees. Simulation of PXRD powder pattern was calculated using Visualization for Electronic and Structural Analysis (VESTA) v. 3.4.4 from the CIF file of the respective MOF retrieved from the respective reference.

Nitrogen gas adsorption isotherms were performed on a Quantachrome NOVA 2200e surface area and porosity analyzer at 77 K. Prior to the tests, all samples were thermally activated overnight at 100 °C under 0.01 mmHg. Helium gas was used to measure the dead space volume prior to measurements. Brunauer–Emmett–Teller (BET) surface areas were determined by linear least-square fitting of the BET plot, the upper working limits were provided by the Rouquerol analysis. Following the procedure by Snurr *et al*., the volume of the monolayer, BET surface areas, and C-constant were determined for the MOFs^26^.

Thermogravimetric analysis (TGA) was performed using a TA Instruments Q5000 from room temperature to 600 °C at 10 °C/min under a continuous nitrogen flow. Test results were analyzed using Universal Analysis Software.

To get insights into the compatibility of different phases, the characteristics of the rubber fracture surfaces were observed via scanning electron microscopy (SEM) using a Thermo Scientific™ Scios™ DualBeam™ operated at 5 kV. All samples were first fractured in a consistent manner after soaking in liquid nitrogen for 5 minutes. Fractured sample surfaces were sputter-coated with iridium prior to SEM imaging.

The particle size of MOF was measured using a Dynamic Light Scattering (DLS) analyzer- Malvern Zetasizer Nano ZS. A sample of 0.100 mg was dispersed in methanol and sonicated for 1 h prior to analysis.

### Preparation of MOFs

MOFs were prepared according to previously reported methods: **Ti-MIL-125**^13^, **Ti-MIL-125-NH**_**2**_^16^, **Zr-MOF-808**^17^, **Zr-UiO-66**^14^, **Zr-UiO-66-NH**_**2**_^14^, **Cr-MIL-101**^15^, **Cu-HKUST-1**^19^, **Zn-ZIF-8 (Zn)**^18^, and **Al-MIL-53**^12^. Isolation was performed by vacuum filtration after MOF samples cooled to room temperature naturally. Samples were washed with DMF (3×, ~25 mL) followed by CHCl_3_ (3×, ~25 mL). Samples were then generally immersed in 25 mL of MeOH for 2 d in a desiccator, replacing solvent through this time (6×). Residual MeOH was decanted off and finally dried under dynamic vacuum (0.01 mmHg) at 120 °C for 16 h to yield activated samples and stored under Ar atmosphere until further studies. Characterization results are consistent with the corresponding literature data, confirming proper synthesis and activation procedure.

### Water uptake studies

Gravimetric uptake studies were performed in a home-built environmental chamber using a TaoTronics ultrasonic cool mist humidifier charged with Ultrapure water (Milli-Q system) and using joule heating as the heat source for relative humidity (RH) control. A loose grain powder bed was used to simulate working conditions. Fully activated MOF powder was placed flat inside polypropylene beakers with a flat bottom and parallel sidewalls. The temperature and RH were respectively controlled at 295 K and 70%, unless specified otherwise, at ambient pressure. The RH and temperatures of the system were verified using a high-accuracy secondary thermocouple and humidity sensor that shows variations of less than 5% RH and 2 °C. A calibrated balance was used to record masses at predetermined time intervals.

Measurements were first performed for several days to assess ideal experimental conditions. A loose grain powder bed geometry with a fixed packing porosity (~0.65–0.7) and thickness was changed from 1 mm to 10 mm. The porosity of the MOF powder bed (*φ*) was calculated from the following equation:

3 $$\varphi =1-\frac{\,{\rho }_{AppMOF}}{{\rho }_{PowderPart}}$$

where *ρ*_*AppMOF*_ is the apparent density of the MOF and *ρ*_*PowderPart*_ is powder particle density which is determined from pycnometry and BET determined pore volumes. Kim *et al*. determined, in the case of **Zr-MOF-801**, that a porosity above 0.5 as necessary to ensure molecular and Knudsen diffusion behavior is avoided^7^. We expect similar results in the case of MOFs investigated here, for they have similar crystallite sizes.

Prior to water desorption, MOFs were allowed to saturate with water at 70% RH and 22 °C for at least 24 h to minimize measurement uncertainties, which could potentially arise from not being at equilibrium. A Lindberg Blue-M vacuum oven operated at ambient pressure and 30% RH to simulate environmental conditions would be set at the target temperature. Samples were placed into the oven and weigh periodically. The RH and temperatures of the system were verified using a high-accuracy secondary thermocouple and humidity sensor that shows variations of less than 6% RH and 2 °C.

Water cycle stability and recovery, the working capacity (g_h2o_ kg^−1^_MOF_), was performed in triplicate over 10 cycles on a 100 mg scale. Adsorption cycles were performed at 70% RH over 24 h. Desorption cycles were performed at 60 °C for 24 h under ambient RH (~30%). Working capacity in the context of this study is defined as the amount of water vapor that is desorbed.

Kinetic measurements were performed through the course of 4000 min. To minimize Knudsen diffusion and potential thermal effects, measurements were first taken of thickness and packing porosity of 1 mm and 0.65, respectively. The adsorption kinetics for 70% RH and 22 °C was performed in triplicate to ensure reproducibility (Fig. S38) — all remaining kinetics plots as a single measurement. The effect of RH was studied by maintaining the temperature at 22 °C and fluctuation in the RH from 50–70% RH. Thermal effects of adsorption were studied by maintaining RH at 70% and varying the temperature from 22 to 27 and 35 °C. The effect of thickness was studied at 1, 2, 5, and 10 mm. Desorption studies were performed at 60, 50, and 40 °C.

## Supplementary information

Supplementary Information.

## Data Availability

All data generated or analyzed during this study are included in this published article (and its Supplementary Information file).
