# Supplementary material for: Reversible Atmospheric Water Harvesting Using Metal-Organic Frameworks
Source: Sci Rep. 2020 Jan 30;10:1492. doi: 10.1038/s41598-020-58405-9 (PMC6992632; doi:10.1038/s41598-020-58405-9)
Supplement: Supplementary file 1 — Supplementary Information. [file 41598_2020_58405_MOESM1_ESM.docx]

*Supplementary Information of*

**Reversible Atomospheric Water Harevesting Using Metal-Organic Frameworks**

Matthew W. Logan, Spencer Langevin, and Zhiyong Xia*

Research and Exploratory Development Department, The Johns Hopkins University Applied Physics Laboratory, Laurel, MD 20723, United States

| **Correspondence Address** |
| --- |
| *Zhiyong Xia, Ph.D.  Research and Exploratory Development Department  Applied Physics Laboratory  Johns Hopkins University  11100 Johns Hopkins Road  Laurel, MD 20723 (USA)  Tel: (+1)-240-228-9694  Email: *Zhiyong.Xia@jhuapl.edu* |

**Contents**

S1. Powder X-ray Diffraction S3

S2. N_2_ Gas Adsorption Isotherms S8

S3. Thermal Analysis S13

S4. Scanning Electron Microscopy S18

S5. Dynamic Light Scattering S23

S6. Kinetic Modeling S28

S7. References S45

***Section S1*. Powder X-ray Diffractograms (PXRD)**

**Figure S1.** PXRD of as-synthesized **Ti-MIL-125** (red) and **Ti-MIL-125** (blue) after 10 adsorption-desorption cycles with corresponding simulated **Ti-MIL-125** (black).

**Figure S2.** PXRD of as-synthesized **Ti-MIL-125-NH_2_** (red) and **Ti-MIL-125-NH_2_** (blue) after 10 adsorption-desorption cycles with corresponding simulated **Ti-MIL-125-NH_2_** (black).

**Figure S3.** PXRD of as-synthesized **Zr-UiO-66** (red) and **Zr-UiO-66** (blue) after 10 adsorption-desorption cycles with corresponding simulated **Zr-UiO-66** (black).

**Figure S4.** PXRD of as-synthesized **Zr-UiO-66-NH_2_** (red) and **Zr-UiO-66-NH_2_** (blue) after 10 adsorption-desorption cycles with corresponding simulated **Zr-UiO-66-NH_2_** (black).

**Figure S5.** PXRD of as-synthesized **Zr-MOF-808** (red) and **Zr-MOF-808** (blue) after 10 adsorption-desorption cycles with corresponding simulated **Zr-MOF-808** (black).

**Figure S6.** PXRD of as-synthesized **Cr-MIL-101** (red) and **Cr-MIL-101** (blue) after 10 adsorption-desorption cycles with corresponding simulated **Cr-MIL-101** (black).

**Figure S7.** PXRD of as-synthesized **Al-MIL-53** (red) and **Al-MIL-53** (blue) after 10 adsorption-desorption cycles with corresponding simulated **Al-MIL-53** (black).

**Figure S8.** PXRD of as-synthesized **Cu-HKUST-1** (red) and **Cu-HKUST-1** (blue) after 10 adsorption-desorption cycles with corresponding simulated **Cu-HKUST-1** (black).

**Figure S9.** PXRD of as-synthesized **Zn-ZIF-8** (red) and **Zn-ZIF-8** (blue) after 10 adsorption-desorption cycles with corresponding simulated **Zn-ZIF-8** (black).

***Section S2*. Gas Adsorption**

**Figure S10.** Linear N_2_ gas (77 K, *p*_0_ = 760 torr) adsorption-desorption isotherm for **Ti-MIL-125**. Closed circles represent adsorption, open circles, desorption.

**Figure S11.** Linear N_2_ gas (77 K, *p*_0_ = 760 torr) adsorption-desorption isotherm for **Ti-MIL-125-NH_2_**. Closed circles represent adsorption, open circles, desorption.

**Figure S12.** Linear N_2_ gas (77 K, *p*_0_ = 760 torr) adsorption-desorption isotherm for **Zr-UiO-66**. Closed circles represent adsorption, open circles, desorption.

**Figure S13.** Linear N_2_ gas (77 K, *p*_0_ = 760 torr) adsorption-desorption isotherm for **Zr-UiO-66-NH_2_**. Closed circles represent adsorption, open circles, desorption.

**Figure S14.** Linear N_2_ gas (77 K, *p*_0_ = 760 torr) adsorption-desorption isotherm for **Zr-MOF-808**. Closed circles represent adsorption, open circles, desorption.

**Figure S15.** Linear N_2_ gas (77 K, *p*_0_ = 760 torr) adsorption-desorption isotherm for **Cr-MIL-101**. Closed circles represent adsorption, open circles, desorption.

**Figure S16.** Linear N_2_ gas (77 K, *p*_0_ = 760 torr) adsorption-desorption isotherm for **Al-MIL-53**. Closed circles represent adsorption, open circles, desorption.

**Figure S17.** Linear N_2_ gas (77 K, *p*_0_ = 760 torr) adsorption-desorption isotherm for **Cu-HKUST-1**. Closed circles represent adsorption, open circles, desorption.

**Figure S18.** Linear N_2_ gas (77 K, *p*_0_ = 760 torr) adsorption-desorption isotherm for **Zn-ZIF-8**. Closed circles represent adsorption, open circles, desorption.

***Section S3*. Thermal Analysis – Thermal Gravimetric Analysis (TGA)**

**Figure S19.** TGA for **Ti-MIL-125** under N_2_ gas atmosphere (10 °C min^-1^).

**Figure S20.** TGA for **Ti-MIL-125-NH_2_** under N_2_ gas atmosphere (10 °C min^-1^).

**Figure S21.** TGA for **Zr-UiO-66** under N_2_ gas atmosphere (10 °C min^-1^).

**Figure S22.** TGA for **Zr-UiO-66-NH_2_** under N_2_ gas atmosphere (10 °C min^-1^).

**Figure S23.** TGA for **Zr-MOF-808** under N_2_ gas atmosphere (10 °C min^-1^).

**Figure S24.** TGA for **Cr-MIL-101** under N_2_ gas atmosphere (10 °C min^-1^).

**Figure S25.** TGA for **Al-MIL-53** under N_2_ gas atmosphere (10 °C min^-1^).

**Figure S26.** TGA for **Cu-HKUST-1** under N_2_ gas atmosphere (10 °C min^-1^).

**Figure S27.** TGA for **Zn-ZIF-8** under N_2_ gas atmosphere (10 °C min^-1^).

***Section S4*. Scanning Electron Microscopy (SEM)**

**
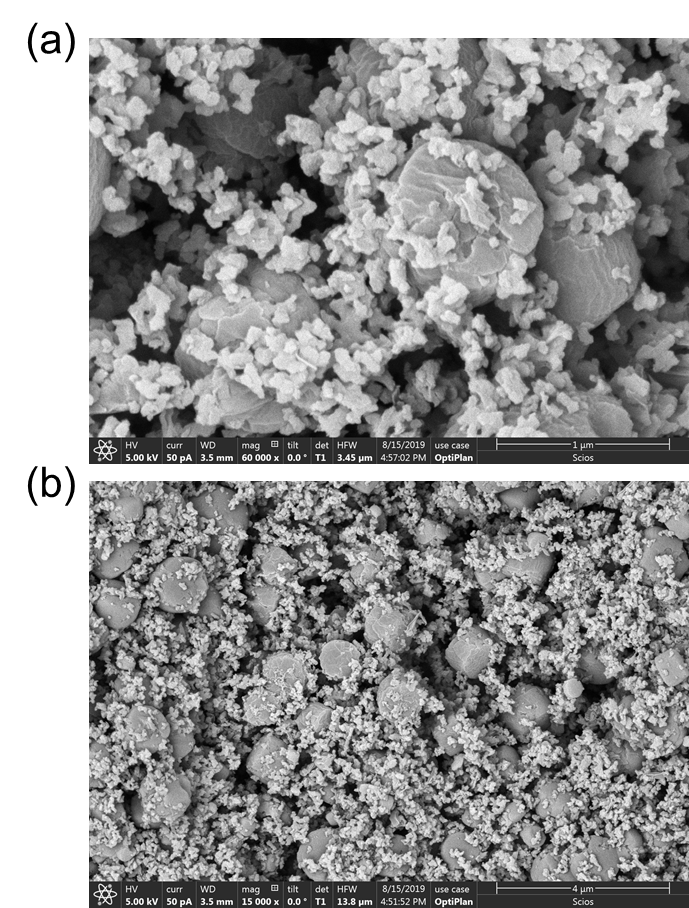
**

**Figure S28.** Scanning electron microscopy of **Ti-MIL-125**. The scale is indicated on images.


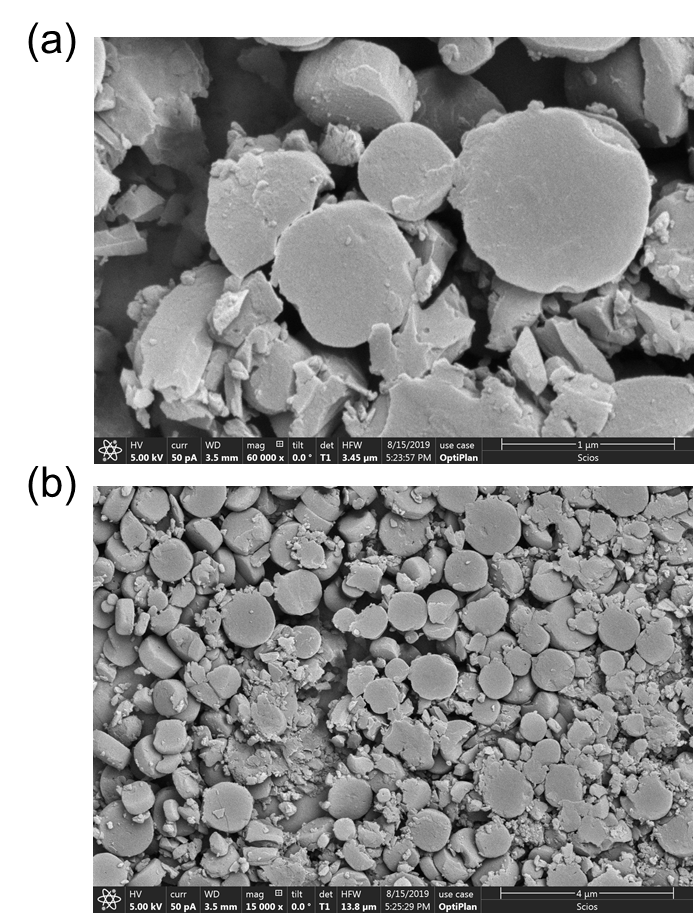


**Figure S29.** Scanning electron microscopy of **Ti-MIL-125-NH_2_**. The scale is indicated on images.


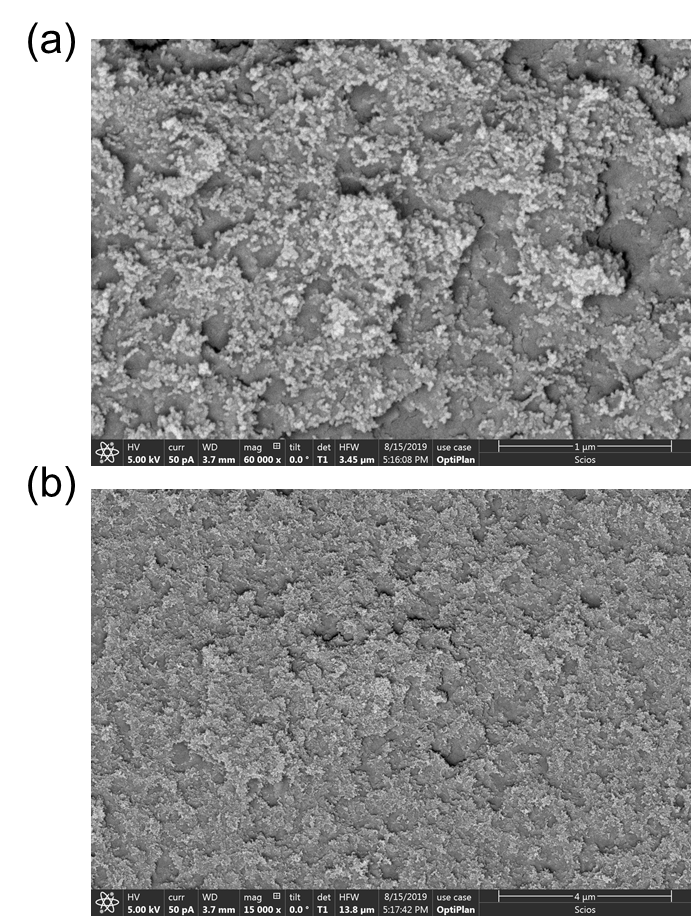


**Figure S30.** Scanning electron microscopy of **Zr-UiO-66-NH_2_**. The scale is indicated on images.


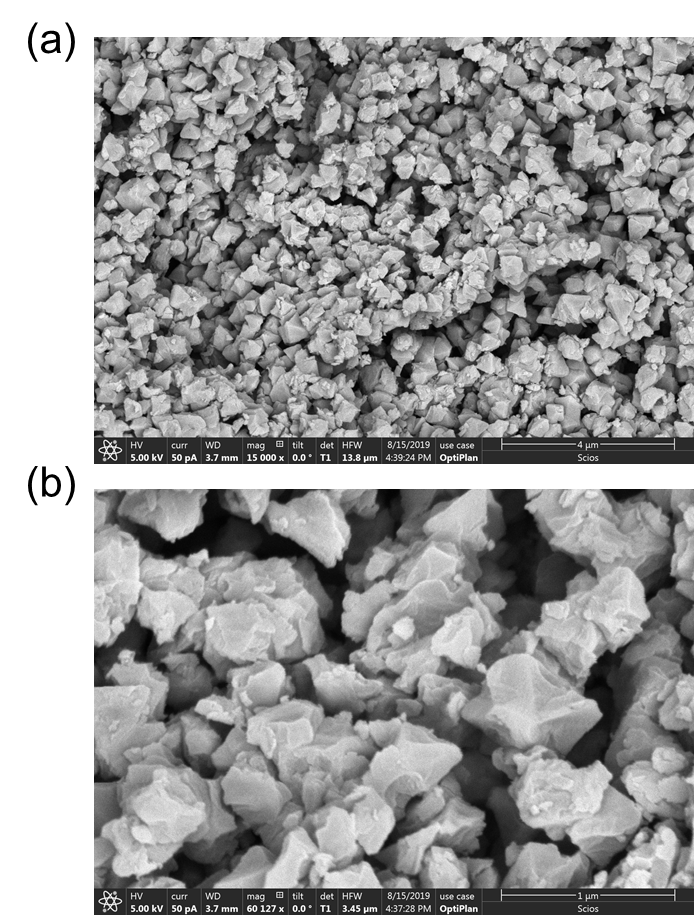


**Figure S31.** Scanning electron microscopy of **Zr-MOF-808**. The scale is indicated on images.


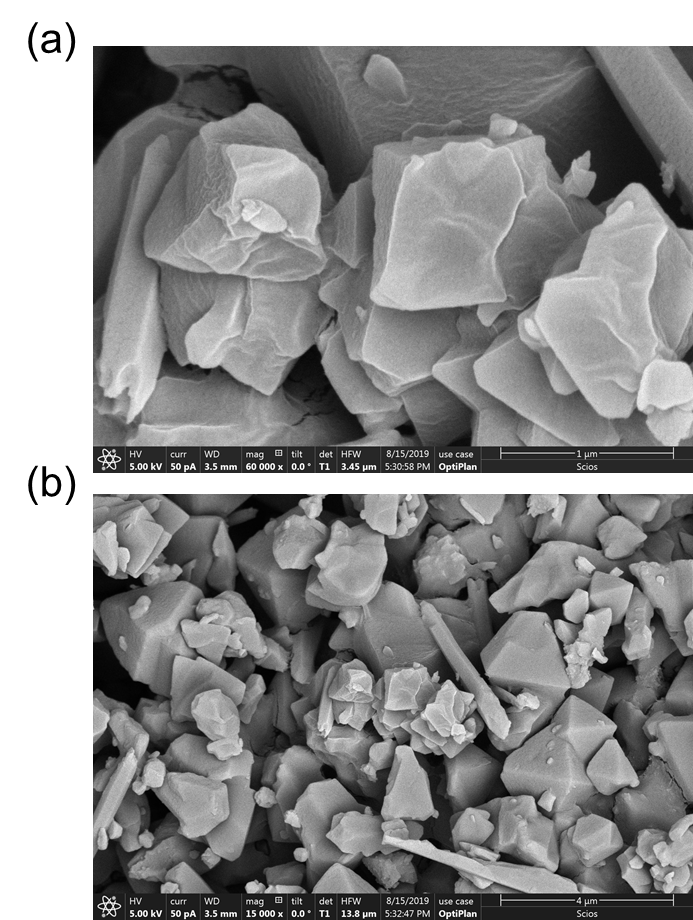


**Figure S32.** Scanning electron microscopy of **Cr-MIL-101**. The scale is indicated on images.

***Section S5*. Dynamic Light Scattering (DLS)**


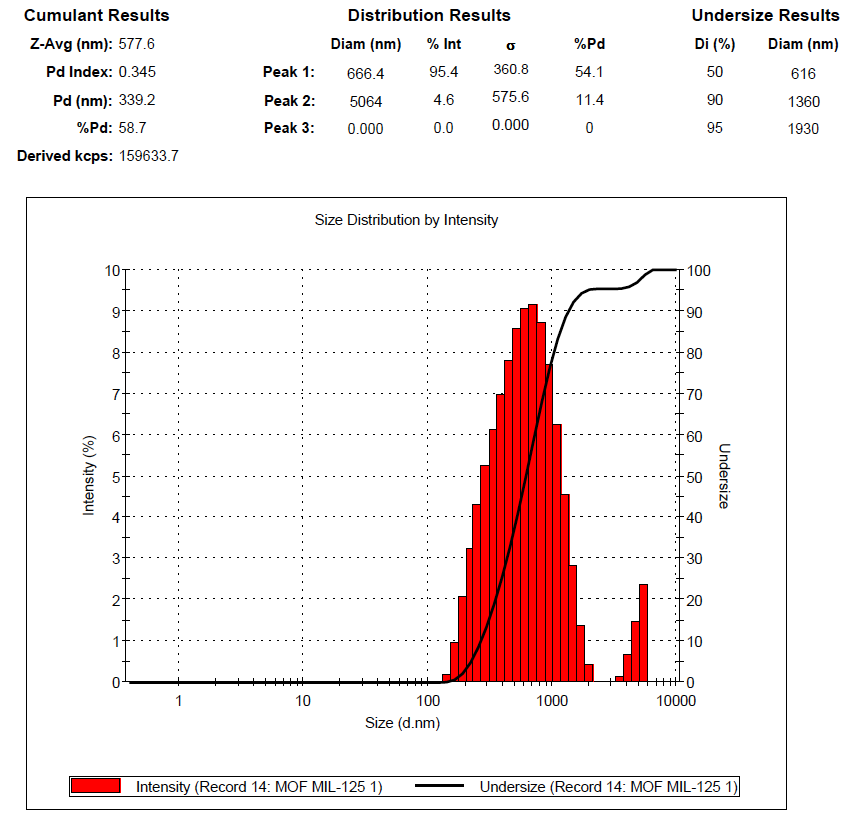


**Figure S33.** Particle size distribution profiles obtained by DLS of the **Ti-MIL-125**.


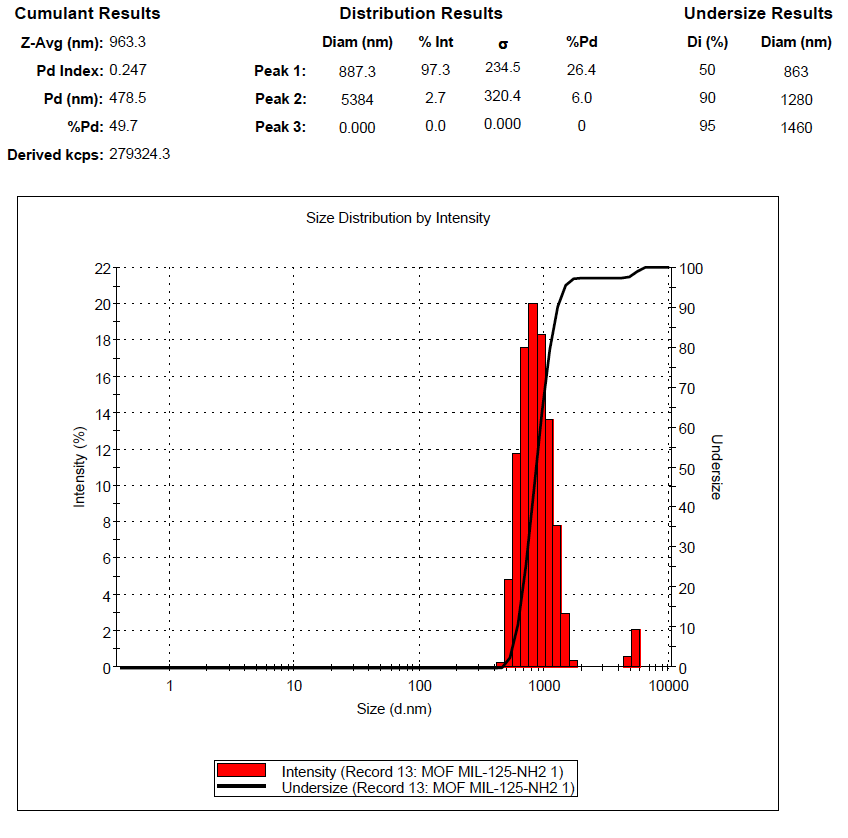


**Figure S34.** Particle size distribution profiles obtained by DLS of **Ti-MIL-125-NH_2_**.


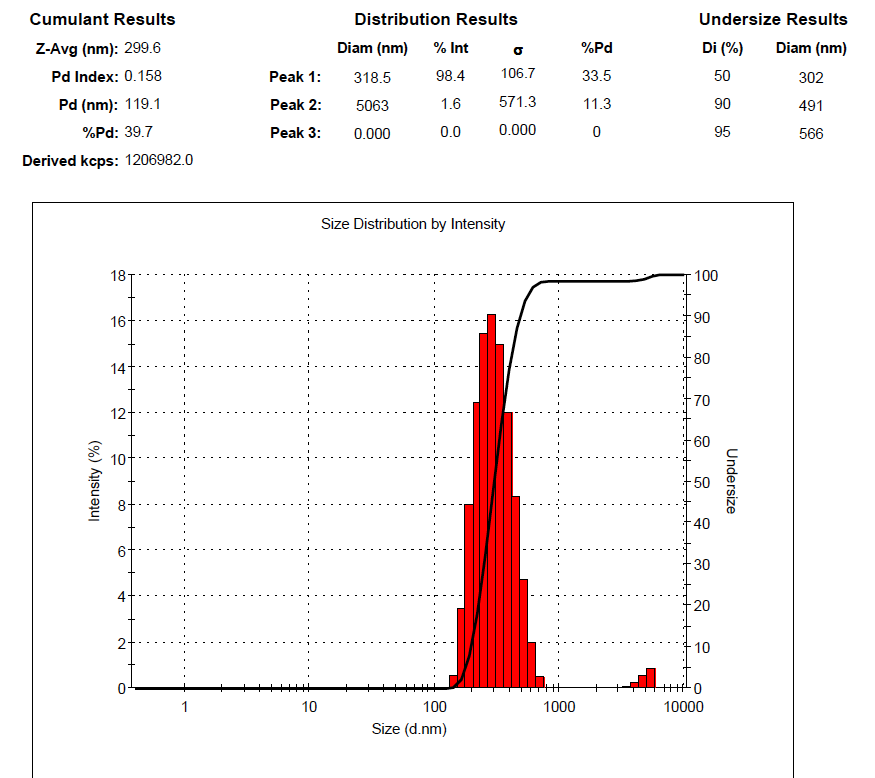


**Figure S35.** Particle size distribution profiles obtained by DLS of **Zr-UiO-66-NH_2_**


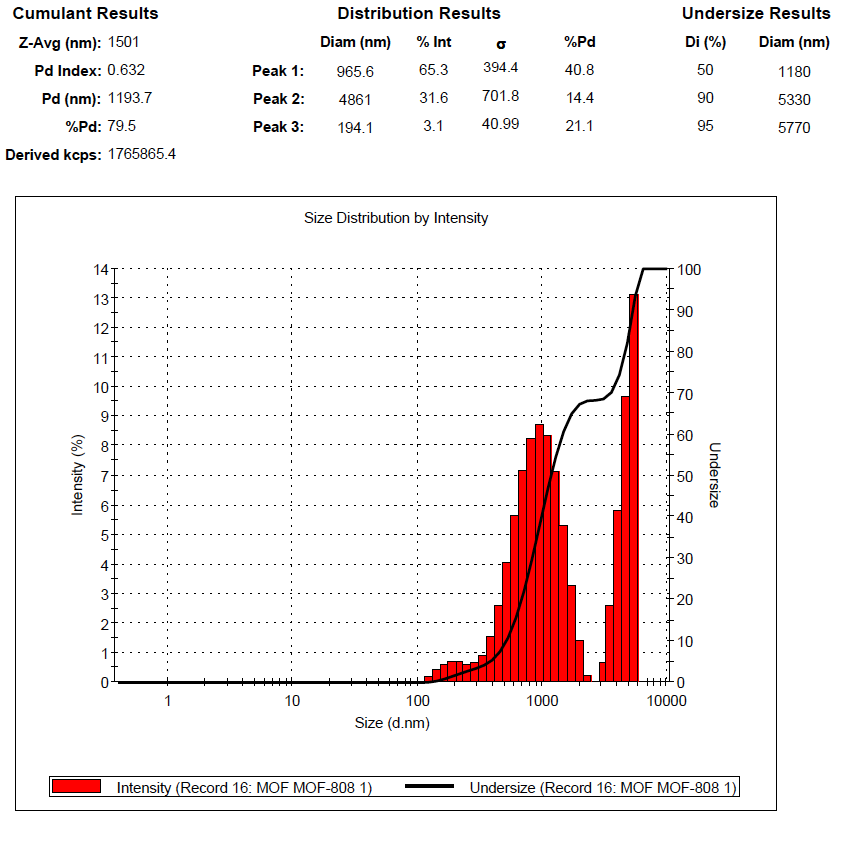


**Figure S36.** Particle size distribution profiles obtained by DLS of **Zr-MOF-808**.


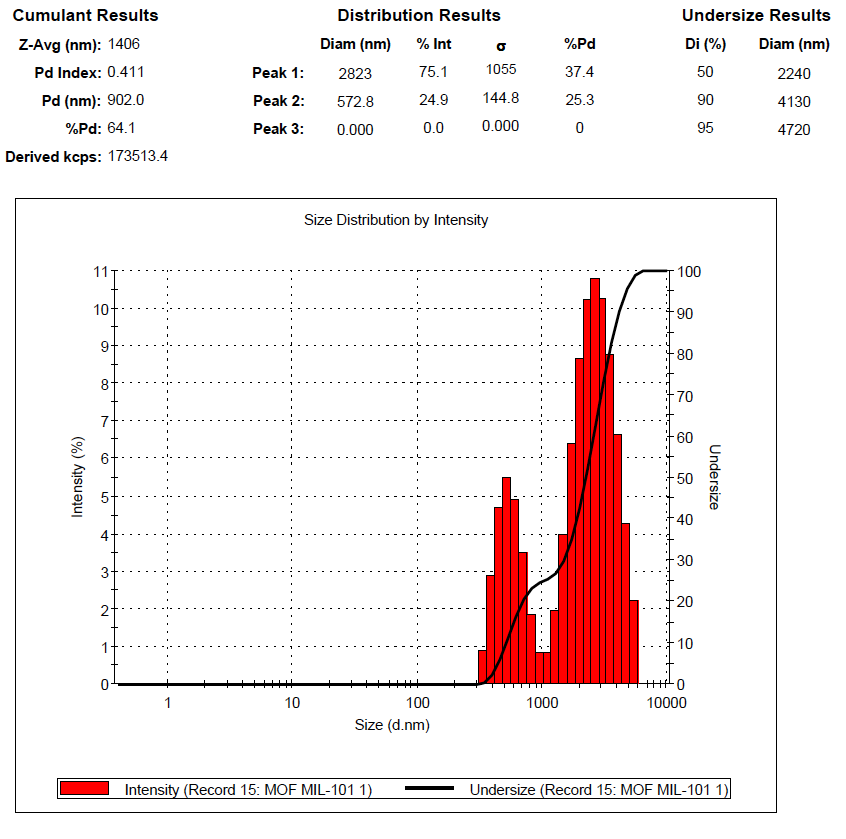


**Figure S37.** Particle size distribution profiles obtained by DLS of **Cr-MIL-101**.

***Section S6*. Kinetic Modeling**

**Figure S38.** Reproducibility of the dynamic water vapor adsorption in a loose power bed geometry of **Ti-MIL-125** (black), **Ti-MIL-125-NH_2_** (orange), **Cr-MIL-101** (blue), **Zr-MOF-808** (green), and **Zr-UiO-66-NH_2_** (yellow) at 22 °C with a sample depth of 1 mm, 70% RH, and packing porosities of ~0.65-0.7. Error bars represent ± one standard deviation of runs ran in triplicate on fully activated samples.

**Figure S39.** Dynamic water vapor adsorption in a loose power bed geometry of **Ti-MIL-125** (black), **Ti-MIL-125-NH_2_** (orange), **Cr-MIL-101** (blue), **Zr-MOF-808** (green), and **Zr-UiO-66-NH_2_** (yellow) at 22 °C with a sample depth of 1 mm, 70% RH, and packing porosities of ~0.65-0.7. Experimental results are shown as solid spheres, and LDF fitted plot as a dashed line in representative color.

**Figure S40.** Dynamic water vapor adsorption in a loose power bed geometry of **Ti-MIL-125** (black), **Ti-MIL-125-NH_2_** (orange), **Cr-MIL-101** (blue), **Zr-MOF-808** (green), and **Zr-UiO-66-NH_2_** (yellow) at 22 °C with a sample depth of 1 mm, 60% RH, and packing porosities of ~0.65-0.7. Experimental results are shown as solid spheres, and LDF fitted plot as a dashed line in representative color.

**Figure 41.** Dynamic water vapor adsorption in a loose power bed geometry of **Ti-MIL-125** (black), **Ti-MIL-125-NH_2_** (orange), **Cr-MIL-101** (blue), **Zr-MOF-808** (green), and **Zr-UiO-66-NH_2_** (yellow) at 22 °C with a sample depth of 1 mm, 50% RH, and packing porosities of ~0.65-0.7. Experimental results are shown as solid spheres, and LDF fitted plot as a dashed line in representative color.

**Figure S42.** Dynamic water vapor adsorption in a loose power bed geometry of **Ti-MIL-125** (black), **Ti-MIL-125-NH_2_** (orange), **Cr-MIL-101** (blue), **Zr-MOF-808** (green), and **Zr-UiO-66-NH_2_** (yellow) at 27 °C with a sample depth of 1 mm, 70% RH, and packing porosities of ~0.65-0.7. Experimental results are shown as solid spheres, and LDF fitted plot as a dashed line in representative color.

**Figure S43.** Dynamic water vapor adsorption in a loose power bed geometry of **Ti-MIL-125** (black), **Ti-MIL-125-NH_2_** (orange), **Cr-MIL-101** (blue), **Zr-MOF-808** (green), and **Zr-UiO-66-NH_2_** (yellow) at 35 °C with a sample depth of 1 mm, 70% RH, and packing porosities of ~0.65-0.7. Experimental results are shown as solid spheres, and LDF fitted plot as a dashed line in representative color.

**Figure S44.** Dynamic water vapor adsorption in a loose power bed geometry of **Ti-MIL-125** (black), **Ti-MIL-125-NH_2_** (orange), **Cr-MIL-101** (blue), **Zr-MOF-808** (green), and **Zr-UiO-66-NH_2_** (yellow) at 22 °C with a sample depth of 2 mm, 70% RH, and packing porosities of ~0.65-0.7. Experimental results are shown as solid spheres, and LDF fitted plot as a dashed line in representative color.

**Figure S45.** Dynamic water vapor adsorption in a loose power bed geometry of **Cr-MIL-101** at 22 °C with a sample depth of 1 (blue), 2 (green), 5 (yellow) and 10 (red) mm at 70% RH. Experimental results are shown as solid spheres, LDF fitted plot as dashed line, and pseudo-zero order fitted as a solid line in representative color.

**Figure S46.** Dynamic water vapor desorption in a loose power bed geometry of **Ti-MIL-125** (black), **Ti-MIL-125-NH_2_** (orange), **Cr-MIL-101** (blue), **Zr-MOF-808** (green), and **Zr-UiO-66-NH_2_** (yellow) at 40 °C with a sample depth of 1 mm, 30% RH, and packing porosities of ~0.65-0.7. Experimental results are shown as solid spheres, and LDF fitted plot as a dashed line in representative color.

**Figure S47.** Dynamic water vapor desorption in a loose power bed geometry of **Ti-MIL-125** (black), **Ti-MIL-125-NH_2_** (orange), **Cr-MIL-101** (blue), **Zr-MOF-808** (green), and **Zr-UiO-66-NH_2_** (yellow) at 50 °C with a sample depth of 1 mm, 30% RH, and packing porosities of ~0.65-0.7. Experimental results are shown as solid spheres, and LDF fitted plot as a dashed line in representative color.

**Figure S48.** Dynamic water vapor desorption in a loose power bed geometry of **Ti-MIL-125** (black), **Ti-MIL-125-NH_2_** (orange), **Cr-MIL-101** (blue), **Zr-MOF-808** (green), and **Zr-UiO-66-NH_2_** (yellow) at 60 °C with a sample depth of 1 mm, 30% RH, and packing porosities of ~0.65-0.7. Experimental results are shown as solid spheres, and LDF fitted plot as a dashed line in representative color.

**Figure S49.** The Arrhenius plot showing the dependence of the adsorption on temperature for water vapor adsorption by MOFs at 70% RH and sample depth of 1 mm of **Ti-MIL-125** (black), **Ti-MIL-125-NH_2_** (orange), **Cr-MIL-101** (blue), **Zr-MOF-808** (green), and **Zr-UiO-66-NH_2_** (yellow)

Table S1. Adsorption experimental results of the kinetic mass-transfer, diffusion coefficient parameters, and diffusional activation energy of water vapor on tested MOFs at 70% RH^a^

| MOF | Uptake capacity (g_water_ kg_MOF_-1) | | α*c*  (RH, %) | Ref. |
| --- | --- | --- | --- | --- |
|  | Gravimetric*a* | Volumetric*b* |  |  |
| **Ti-MIL-125** | 323 | 360 | 25 | S^[[1]](#endnote-2)^ |
| **Ti-MIL-125-NH_2_** | 413 | 370 | 20 | S^[[2]](#endnote-3)^ |
| **Zr-UiO-66** | 347 | 360 | 30 | S^[[3]](#endnote-4)^ |
| **Zr-UiO-66-NH_2_** | 364 | 340 | 16 | S^[[4]](#endnote-5)^ |
| **Zr-MOF-808** | 732 | 730 | 36 | S^[[5]](#endnote-6)^ |
| **Cr-MIL-101** | 1235 | 1280 | 46 | S^[[6]](#endnote-7)^ |
| **Cu-HKUST-1** | 486 | 510 | 20 | S3 |
| **Al-MIL-53** | 13 | 90 | 14 | S^[[7]](#endnote-8)^ |
| **Zn-ZIF-8** | 15 | 10 | 80 | S6 |

*^a^*Working capacity was determined gravimetrically by the difference in the amount of water desorbed and adsorbed during water cycle stability and recovery studies. *^b^*Volumetric capacity reported previously in the corresponding reference, determined by water vapor isotherm. *^c^* Inflex the determined by water vapor adsorption isotherm.

Table S2. LDA fitting of experimental results of variable temperature water vapor adsorptions on tested MOFs.*^a^*

| MOF | 22 °C | | | 27 °C | | | 35 °C | | | E_a_  (kj mol-1) | R2_adj_ |
| --- | --- | --- | --- | --- | --- | --- | --- | --- | --- | --- | --- |
|  | *k* (min-1) | R2_adj_ | R_0_ (g kg-1 min-1) | *k* (min-1) | R2_adj_ | R_0_ (g kg-1 min-1) | *k* (min-1) | R2_adj_ | R_0_ (g kg-1 min-1) |  |  |
| Ti-MIL-125 | 3.02 × 10-2 | 0.974 | 8.65 | 3.21 × 10-2 | 0.975 | 9.21 | 3.56 × 10-2 | 0.972 | 1.02 × 10 | 9.62 | 0.999 |
| Ti-MIL-125-NH_2_ | 4.42 × 10-2 | 0.986 | 1.98 × 10 | 4.13 × 10-2 | 0.999 | 1.85 × 10 | 3.84 × 10-2 | 0.987 | 1.72 × 10 | -8.17 | 0.988 |
| Zr-UiO-66-NH_2_ | 4.52 × 10-2 | 0.996 | 1.72 × 10 | 3.24 × 10-2 | 0.986 | 1.23 × 10 | 2.58 × 10-2 | 0.956 | 9.82 | -31.7 | 0.949 |
| Zr-MOF-808 | 7.20 × 10-2 | 0.999 | 4.74 × 10 | 5.16 × 10-2 | 0.988 | 3.40 × 10 | 3.95 × 10-2 | 0.970 | 2.60 × 10 | -34.1 | 0.966 |
| Cr-MIL-101 | 6.35 × 10-3 | 0.996 | 7.97 | 7.10 × 10-3 | 0.999 | 8.91 | 7.78 × 10-3 | 0.998 | 9.87 | 12.3 | 0.978 |

***^a^***Loose powder bed samples with a packing porosity of ~ 0.65-0.7 and sample depths of 1 mm at 22 **°**C. The average particle size was used to calculate the diffusional coefficient.

Table S3. LDA fitting of experimental results of variable temperature water vapor desorption on tested MOFs.*^a^*

| MOF | 40 °C | | | 50 °C | | | 60 °C | | |
| --- | --- | --- | --- | --- | --- | --- | --- | --- | --- |
|  | *k* (min-1) | R2_adj_ | R_0_ (g kg-1 min-1) | *k* (min-1) | R2_adj_ | R_0_ (g kg-1 min-1) | *k* (min-1) | R2_adj_ | R_0_ (g kg-1 min-1) |
| **Ti-MIL-125** | 3.78 × 10-3 | 0.999 | 1.08 | 4.75 × 10-2 | 0.975 | 1.36 × 10 | 8.71 × 10-2 | 0.993 | 2.50 × 10 |
| **Ti-MIL-125-NH_2_** | 1.41 × 10-3 | 0.991 | 6.32 × 10-1 | 1.53 × 10-2 | 0.976 | 6.86 | 3.37 × 10-2 | 0.996 | 1.67 × 10 |
| **Zr-UiO-66-NH_2_** | 6.93 × 10-3 | 0.956 | 2.64 | 2.91 × 10-2 | 0.976 | 1.11 × 10 | 4.73 × 10-2 | 0.990 | 1.80 × 10 |
| **Zr-MOF-808** | 1.64 × 10-2 | 0.988 | 1.08 × 10 | 7.90 × 10-2 | 0.994 | 5.21 × 10 | 1.13 × 10-1 | 0.993 | 7.45 × 10 |
| **Cr-MIL-101** | 8.21 × 10-3 | 0.971 | 1.03 × 10 | 1.21 × 10-2 | 0.999 | 1.52 × 10 | 2.13 × 10-2 | 0.982 | 2.67 × 10 |

***^a^***Loose powder bed samples with a packing porosity of ~ 0.65-0.7 and sample depths of 1 mm at 22 **°**C. The average particle size was used to calculate the diffusional coefficient.

Table S4. LDA fitting of experimental results of variable RH water vapor adsorptions on tested MOFs.*^a^*

| MOF | 50% RH | | | 60% RH | | | 70% RH | | | |
| --- | --- | --- | --- | --- | --- | --- | --- | --- | --- | --- |
|  | *k* (min-1) | R2_adj_ | *D_H2O_* (cm 2 min-1) | *k* (min-1) | R2_adj_ | *D_H2O_* (cm 2 min-1) | *k* (min-1) | R2_adj_ | *D_H2O_* (cm 2 min-1) | |
| **Ti-MIL-125** | 3.50 × 10-2 | 0.978 | 5.55 × 10-4 | 3.95× 10-2 | 0.976 | 6.10 × 10-4 | 3.02 × 10-2 | 0.974 | 6.72 × 10-4 |  |
| **Ti-MIL-125-NH_2_** | 4.10 × 10-2 | 0.988 | 2.33 × 10-3 | 4.34 × 10-2 | 0.996 | 2.52 × 10-3 | 4.42 × 10-2 | 0.986 | 2.74 × 10-3 |  |
| **Zr-UiO-66-NH_2_** | 2.27 × 10-2 | 0.991 | 1.03 × 10-4 | 3.65 × 10-2 | 0.988 | 2.36 × 10-4 | 4.52 × 10-2 | 0.996 | 2.70 × 10-4 |  |
| **Zr-MOF-808** | 4.25 × 10-2 | 0.997 | 7.22 × 10-3 | 5.05 × 10-2 | 0.998 | 8.47 × 10-3 | 7.20 × 10-2 | 0.999 | 1.08 × 10-2 |  |
| **Cr-MIL-101** | 4.02 × 10-3 | 0.981 | 3.90 × 10-4 | 5.31 × 10-3 | 0.989 | 5.86 × 10-4 | 6.35 × 10-3 | 0.996 | 8.37 × 10-4 |  |

*^a^*The average crystallite size was used to calculate the intracrystalline diffusivity coefficient.

Table S5. LDA fitting of experimental water vapor adsorption results of variable sample depth on tested MOFs at 22 °C.*^a^*

| MOF (Sample depth in mm) | R_0_ (g kg-1 min-1) | *k* (min-1) | R2_adj_ |
| --- | --- | --- | --- |
| **Ti-MIL-125 (2)** | 7.11 | 2.48 × 10-2 | 0.979 |
| **Ti-MIL-125-NH_2_ (2)** | 7.58 | 1.69 × 10-2 | 0.976 |
| **Zr-UiO-66-NH_2_ (2)** | 9.68 | 2.54 × 10-2 | 0.994 |
| **Zr-MOF-808 (2)** | 7.45 | 1.13 × 10-2 | 0.981 |
| **Cr-MIL-101 (2)** | 6.69 | 5.33 × 10-3 | 0.990 |
| **Cr-MIL-101 (1)** | 7.97 | 6.35 × 10-3 | 0.996 |
| **Cr-MIL-101(5) *a*** | 1.32 | 1.09 × 10-3 | 0.999 |
| **Cr-MIL-101(10) *a*** | 7.18 × 10-1 | 5.93 × 10-4 | 0.984 |

*^a^*Fitting performed using zero-order fitting of the linear regime

***Section S8*. References**

1. S. Canivet, J. *et al.* Structure–property relationships of water adsorption in metal–organic frameworks. *New Journal of Chemistry* **38**, 3102-3111 (2014). [↑](#endnote-ref-2)
2. S. Jeremias, F., Lozan, V., Henninger, S. K. & Janiak, C. Programming MOFs for water sorption: amino-functionalized MIL-125 and UiO-66 for heat transformation and heat storage applications. *Dalton Transactions* **42**, 15967-15973 (2013). [↑](#endnote-ref-3)
3. S. Schoenecker, P. M., Carson, C. G., Jasuja, H., Flemming, C. J. & Walton, K. S. Effect of water adsorption on retention of structure and surface area of metal–organic frameworks. *Industrial & Engineering Chemistry Research* **51**, 6513-6519 (2012). [↑](#endnote-ref-4)
4. S. Cmarik, G. E., Kim, M., Cohen, S. M. & Walton, K. S. Tuning the adsorption properties of UiO-66 via ligand functionalization. *Langmuir* **28**, 15606-15613 (2012). [↑](#endnote-ref-5)
5. S. Furukawa, H. *et al.* Water adsorption in porous metal–organic frameworks and related materials. *Journal of the American Chemical Society* **136**, 4369-4381 (2014). [↑](#endnote-ref-6)
6. S. Küsgens, P. *et al.* Characterization of metal-organic frameworks by water adsorption. *Microporous and Mesoporous Materials* **120**, 325-330 (2009). [↑](#endnote-ref-7)
7. S. Canivet, J. *et al.* Structure–property relationships of water adsorption in metal–organic frameworks. *New Journal of Chemistry* **38**, 3102-3111 (2014). [↑](#endnote-ref-8)
